# Heterochromatin-Enriched Assemblies Reveal the Sequence and Organization of the *Drosophila melanogaster* Y Chromosome

**DOI:** 10.1534/genetics.118.301765

**Published:** 2018-11-12

**Authors:** Ching-Ho Chang, Amanda M. Larracuente

**Affiliations:** Department of Biology, University of Rochester, New York 14627

**Keywords:** *Drosophila melanogaster* genome, Y chromosome, long-read assembly, gene duplications, gene conversion, *crystal-Stellate*, Genetics of Sex

## Abstract

Heterochromatic regions of the genome are repeat-rich and poor in protein coding genes, and are therefore underrepresented in even the best genome assemblies. One of the most difficult regions of the genome to assemble are sex-limited chromosomes. The *Drosophila melanogaster* Y chromosome is entirely heterochromatic, yet has wide-ranging effects on male fertility, fitness, and genome-wide gene expression. The genetic basis of this phenotypic variation is difficult to study, in part because we do not know the detailed organization of the Y chromosome. To study Y chromosome organization in *D. melanogaster*, we develop an assembly strategy involving the *in silico* enrichment of heterochromatic long single-molecule reads and use these reads to create targeted *de novo* assemblies of heterochromatic sequences. We assigned contigs to the Y chromosome using Illumina reads to identify male-specific sequences. Our pipeline extends the *D. melanogaster* reference genome by 11.9 Mb, closes 43.8% of the gaps, and improves overall contiguity. The addition of 10.6 MB of Y-linked sequence permitted us to study the organization of repeats and genes along the Y chromosome. We detected a high rate of duplication to the pericentric regions of the Y chromosome from other regions in the genome. Most of these duplicated genes exist in multiple copies. We detail the evolutionary history of one sex-linked gene family, *crystal-Stellate*. While the Y chromosome does not undergo crossing over, we observed high gene conversion rates within and between members of the *crystal-Stellate* gene family, *Su(Ste)*, and *PCKR*, compared to genome-wide estimates. Our results suggest that gene conversion and gene duplication play an important role in the evolution of Y-linked genes.

## Abstract (precis)

Heterochromatic repeat-rich regions are often missing from even the best genome assemblies. Chang and Larracuente designed a de novo assembly strategy to improve the *Drosophila melanogaster* assembly in heterochromatin, extending the reference assembly by 11.9 Mb, including 10.6 Mb from the Y chromosome.....

HETEROCHROMATIC regions of the genome are dense in repetitive elements and rarely undergo recombination via crossing over (Charlesworth *et al.* 1986). While heterochromatin is generally poor in protein coding genes, this compartment of the genome harbors functional elements (Gatti and Pimpinelli 1992) that affect diverse biological processes, including nuclear organization (Csink and Henikoff 1996), chromosome pairing and segregation (Dernburg *et al.* 1996; McKee *et al.* 2000; Rošić *et al.* 2014), and speciation (*e.g.*, Bayes and Malik 2009; Ferree and Barbash 2009; Cattani and Presgraves 2012). The functionally relevant sequences are mostly unknown, in part because it is difficult to sequence and assemble repeat-rich heterochromatic sequences. These sequences can be unstable in cloning vectors and/or toxic to *Escherichia coli* cells (Carlson and Brutlag 1977; Lohe and Brutlag 1987a,b) and thus are underrepresented in clone-based sequencing libraries. Repetitive reads also present a challenge to genome assemblers (Treangen and Salzberg 2011). As a result, many heterochromatic regions of the genome are missing from even the best genome assemblies (Hoskins *et al.* 2002; Carvalho *et al.* 2003). *Drosophila melanogaster* has arguably one of the most contiguous genome assemblies of any metazoan (Chakraborty *et al.* 2016, 2018). However, only ∼143 Mb of the estimated ∼180-Mb haploid genome is assembled into contigs (Hoskins *et al.* 2015). Heterochromatin makes up ∼20% of the female and ∼30% of the male *D. melanogaster* genome (the entire 40-Mb Y chromosome is heterochromatic; Hoskins *et al.* 2002). The latest iteration of the reference genome assembly used BAC-based methods to extend into pericentromeric and telomeric regions, and increased the representation of the Y chromosome over 10-fold—the most recent genome assembly (version 6, R6 hereafter) contains ∼27 Mb of heterochromatin, including ∼4 Mb of Y-linked sequences (Hoskins *et al.* 2015).

The *Drosophila* Y chromosome has been particularly recalcitrant to assembly (Hoskins *et al.* 2015). In addition to problems with cloning and assembly, we expect Y-linked sequences to have 50 and 25% of the autosomal coverage in male and mixed-sex sequencing libraries, respectively. Approximately 80% of the *D. melanogaster* Y chromosome likely consists of tandem repeats (Bonaccorsi and Lohe 1991). There are only ∼20 known Y-linked genes (Carvalho *et al.* 2015), at least six of which are essential for male fertility (Kennison 1981). Despite being poor in protein-coding genes, Y chromosomes can harbor functional variation. Structural variation on the Y chromosome in mammals affects male fertility (Reijo *et al.* 1995; Vogt *et al.* 1996; Sun *et al.* 2000; Repping *et al.* 2003). Similarly, Y-linked genetic variation in *D. melanogaster* has significant effects on male fertility (Chippindale and Rice 2001), including heat-induced male sterility (Rohmer *et al.* 2004). Y-linked genetic variation in *Drosophila* also affects global gene expression (Lemos *et al.* 2008) and chromatin states across the genome (Lemos *et al.* 2010; Brown and Bachtrog 2014 and unpublished data). It is unlikely that this functional variation maps to the few known Y-linked genes because there is very little nucleotide variation in coding regions (Zurovcova and Eanes 1999; Larracuente and Clark 2013). Instead, the Y chromosome may act as a sink for chromatin factors. Variation in the amount of Y-linked heterochromatin may influence the distribution of chromatin modifiers elsewhere in the genome (Dimitri and Pisano 1989; Henikoff 1996; Francisco and Lemos 2014; Brown and Bachtrog 2014 and unpublished data). Without knowing the structure and composition of Y chromosomes, it is difficult to study this phenomenon in detail. Targeted attempts to sequence and assemble the Y chromosome have only had limited success in *Drosophila* (Hoskins *et al.* 2002, 2015; Abad *et al.* 2004; Méndez-Lago *et al.* 2009, 2011; Mahajan *et al.* 2018). Single-molecule long-read sequencing approaches (Branton *et al.* 2008; Eid *et al.* 2009) are improving our ability to assemble repetitive regions of complex genomes (Huddleston *et al.* 2014; Chaisson *et al.* 2015; Chang and Larracuente 2017; Khost *et al.* 2017), including the Y chromosomes of gorilla and human (Tomaszkiewicz *et al.* 2016; Jain *et al.* 2018; Kuderna *et al.* unpublished data). However, these approaches have only resolved relatively small segments of the *Drosophila* Y chromosome (Carvalho *et al.* 2015; Krsticevic *et al.* 2015).

Here, we develop an approach using single-molecule long-read sequencing from Pacific Biosciences (PacBio; Kim *et al.* 2014) to create heterochromatin-enriched genome assemblies and reconcile with whole-genome assemblies. We use this approach to build a new assembly of the *D. melanogaster* genome that fixes current gaps in R6, adds a substantial amount of heterochromatin, and improves the overall contiguity of the genome assembly. Most of the additional sequence in our assembly is Y-linked, allowing us study Y chromosome composition in fine detail. We describe the landscape of transposable elements (TEs), the high rate of Y-linked gene duplication, and patterns of gene conversion among members of Y-linked multicopy gene families.

## Materials and Methods

### Heterochromatin-sensitive assembly

**Figure 1:**
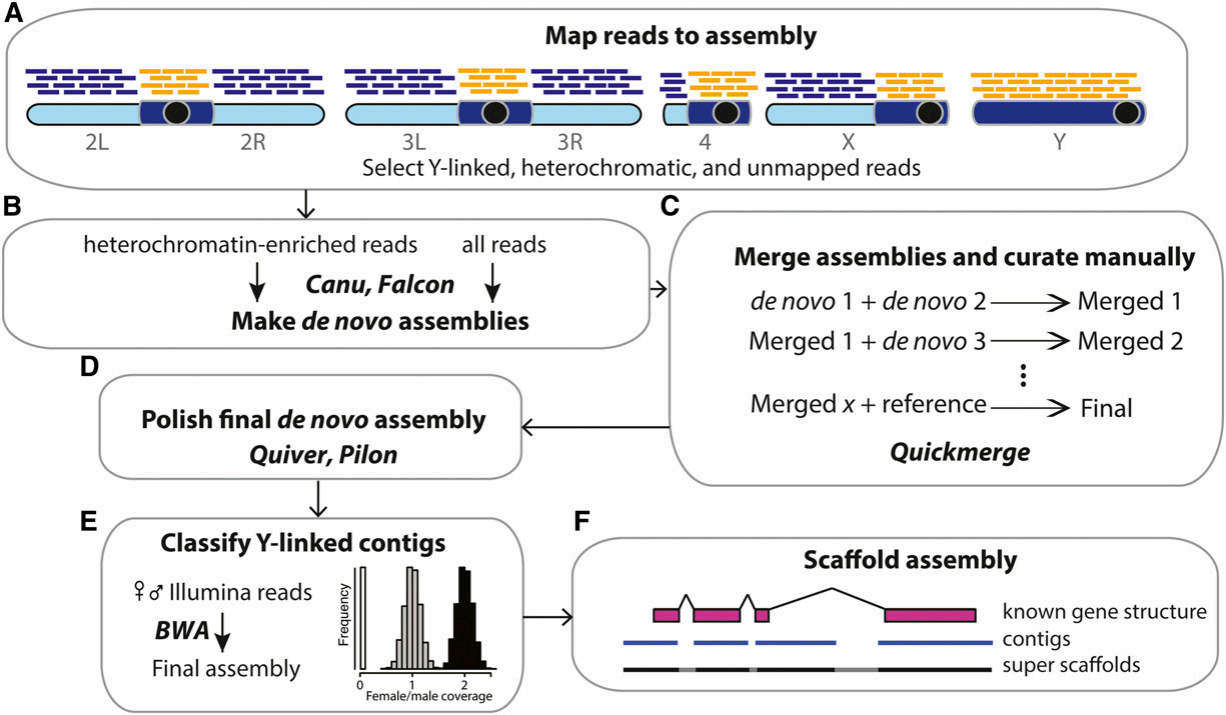
Overview of the heterochromatin-enriched assembly approach. (A) We obtain a set of heterochromatin-enriched PacBio reads by mapping reads to the R6 assembly and retaining reads that map to known pericentric heterochromatin, Y chromosome contigs, or are unmapped (orange lines). (B) We generate separate *de novo* PacBio assemblies for all reads (orange and blue lines) and for heterochromatin-enriched reads (orange lines) with Canu and Falcon. (C) We merge assemblies sequentially using quickmerge to create the final assembly (Table S1). All assemblies were manually inspected and adjusted (see *Materials and Methods*). (D) We polished the final *de novo* assembly with one round of quiver (using raw PacBio reads) and 10 iterations of Pilon (using male Illumina reads). (E) We assign contigs in the final assembly to the X, Y, or autosomes using relative mapping of female-to-male Illumina reads (see *Materials and Methods*). (F) Finally, we join contigs into super scaffolds using exon orientation information from known gene structures.

Our assembly approach is outlined in Figure 1 and Supplemental Material (Figure S1). We used BLASR (v5.1; Chaisson and Tesler 2012) to map PacBio reads [from Kim *et al.* (2014)] to release 6 (R6) of the *D. melanogaster* genome. Both the PacBio sequence reads and the reference genome are from the Iso1 strain. To curate a set of heterochromatin-enriched reads, we extracted any reads that map outside of the major chromosome arms (*i.e.*, 2L, 2R, 3L, 3R, 4, X) and mitochondria, or are unmapped. We took an iterative approach to genome assembly, generating two versions of both the heterochromatin and the whole-genome assemblies, and then reconciling differences between them using quickmerge (Chakraborty *et al.* 2016). For the heterochromatin, we generated *de novo* assemblies with the heterochromatin-enriched reads using Canu v 1.3 (r7740 72c709ef9603fd91273eded19078a51b8e991929; Koren *et al.* 2017; repeat sensitive settings) and Falcon (v0.5; Chin *et al.* 2016; see Supplemental Methods and Table S1). To improve the assembly of the major chromosome arms, we generated *de novo* assemblies with all PacBio reads using Falcon and Canu (Supplemental Methods). We used quickmerge to combine our *de novo* heterochromatin-enriched assemblies with our all-read *de novo* assemblies sequentially, and then with two reference assemblies (R6; Hoskins *et al.* 2015) and a published *de novo* PacBio assembly (Chakraborty *et al.* 2016; Table S1). The detailed Falcon and Canu parameters for each *de novo* assembly and outline of the assembly and reconciliation process are in the Supplemental Methods (Figure S1). We also manually inspected each assembly, paying particular attention to Y-linked genes, where gaps in the assembly can occur because of low-read coverage. We extracted raw or corrected reads from seven Y-linked regions with read coverage <10 and reassembled these manually in Geneious v8.1.6 (Kearse *et al.* 2012). Before attempting to merge any assemblies, we checked that the gene order on all major chromosome arms agreed with R6 and examined the completeness of genes in pericentromeric regions, telomeres, and the Y chromosome. In our final reconciled assembly, we manually adjusted any errors in the *18HT*, *Rsp*, *Sdic*, and *Mst77Y* regions based on their organization in previous studies (Méndez-Lago *et al.* 2009; Krsticevic *et al.* 2015; Clifton *et al.* 2017; Khost *et al.* 2017). We removed redundant contigs using MUMMER implemented in Masurca (v3.2.2; Zimin *et al.* 2017), and polished the resulting assembly using Quiver (SMRT Analysis v2.3.0; Chin *et al.* 2013). To correct base errors in regions with low PacBio coverage, we ran Pilon v1.22 (Walker *et al.* 2014) 10 times with both raw Illumina reads and synthetic reads (Table S2; with parameters “–mindepth 3 -minmq 10–fix bases”). We created two and five scaffolds for the third and Y chromosomes respectively, based on known gene structure. We used MUMMER v3.23 (Kurtz *et al.* 2004) to map our new assembly to the R6 assembly using “nucmer–mum -l 10000 -D 40,” and only reported the one-to-one alignments using “delta-filter −1.” We remapped PacBio reads to this assembly using minimap v2.5-r607 (Li 2016) with parameters “-t 24 -ax map-pb.” We called coverage of uniquely mapped reads using samtools (v1.3 -Q 10; Li *et al.* 2009). To report on the sequence added in our assembly, we define heterochromatic regions based on the coordinates in Hoskins *et al.* (2015) and assume all added sequence beyond these coordinates on major chromosome arms, assigned to the Y chromosome, or on unassigned contigs, is enriched in heterochromatin. We used QUAST v5.0.0 (Mikheenko *et al*. 2018; parameters “-large -fragmented -m 0 -e”) with PacBio reads and Illumina paired-end reads from Wei *et al.* (2018) to evaluate the genome assemblies.

### Identifying Y-linked contigs

We used Illumina reads from male and female PCR-free genomic libraries (Table S2) to identify Y-linked contigs. We mapped the male and female reads separately using BWA (v0.7.15; Li and Durbin 2010) with default settings, and estimated the coverage of uniquely mapped reads per site with samtools (v1.3; -Q 10). We designated contigs with a median female-to-male read ratio of 0 as Y-linked (excluding sites with one or fewer Q >10 reads). To validate the sensitivity and specificity of our methods, we examined our X, Y, and autosome assignments for all 10-kb regions with a known location (only for regions with >1 kb of mappable sites).

### Gene and repeat annotation

We transferred r6.17 FlyBase annotations from the R6 assembly to our final assembly using pBlat (v0.35; https://github.com/icebert/pblat-cluster; Kent 2002) and CrossMap (v0.2.5; Zhao *et al.* 2014). We then used HISAT2 (v2.0.5; Kim *et al.* 2015) to map the male and testes RNA-sequencing reads (Table S2) to the genome based on known splice sites from the new annotation file. We used Stringtie (1.3.3b; Pertea *et al.* 2015) with these mapped reads and the guided annotation file from CrossMap to improve annotations and estimate expression levels. For unknown genes, we searched for homology using NCBI-BLAST against known *D. melanogaster* transcripts sequences (r6.17). To verify misassemblies and duplications, we designed primers to amplify segments of putatively Y-linked contigs/scaffolds with PCR in males and virgin females (Table S3). We also extracted and reverse-transcribed RNA from 3- to 5-day-old testes with TRIzol (ThermoFisher) and M-MLV reverse transcriptase (ThermoFisher), and examined splice sites using RT-PCR (Table S3). We used the gene annotation data to scaffold the assembly.

To annotate repetitive DNA, we used RepeatMasker 4.06 (Smit *et al.* 2013–2015) with Repbase 20150807 and parameters “-species drosophila –s.” We modified scripts from Bailly-Bechet *et al.* (2014) to summarize TEs and other repetitive sequences. We searched for satellites using TRF (v4.09; Benson 1999) with parameters “2 7 7 80 10 100 2000 -ngs -h.”

### Sequence alignments and recombination analyses

We used BLAST v2.2.31+ (Altschul *et al.* 1990) and custom scripts to extract the transcript sequences from the genome. We aligned and manually inspected transcripts using Geneious v8.1.6 (Kearse *et al.* 2012). We constructed phylogenetic trees for regions conserved between members of the *cry-Stellate* family with MrBayes using the autosomal parent gene *Ssl* as an outgroup (GTR + gamma HKY85 model; mcmc ngen = 1,100,000 nchains = 4 temp = 0.2 samplefreq = 200; seed = 20,649). The consensus tree was generated with sumt burnin = 500 with >50% posterior probability. We used the APE phylogenetics package in R (Paradis *et al.* 2004) to plot the tree. We used compute 0.8.4 (Thornton 2003) to calculate Rmin and estimate population recombination rates based on linkage disequilibrium (Hudson 1987). In addition, we estimated gene conversion rates based on gene similarity (Supplemental Methods; Ohta 1984; Rozen *et al.* 2003; Backström *et al.* 2005).

### Data availability

The genome assembly, annotations, and sequence alignments are publicly available at the Dryad Digital Repository (https://doi.org/10.5061/dryad.q91784t). All custom scripts are available in the Dryad file and on GitHub at https://github.com/LarracuenteLab/mel.heterochromatin.Y.assembly. We affirm that all data necessary for confirming the conclusions of the article are present within the article, figures, and tables. Supplemental material (Figures S1–S7, File S1, and Tables S1–S10) available at Figshare: https://doi.org/10.25386/genetics.7294937.

## Results

### Closing gaps in the release six assembly

Major blocks of heterochromatin including the Y chromosome are missing from the latest version of the *D. melanogaster* genome (R6; Hoskins *et al.* 2015). We built a new assembly of the *D. melanogaster* genome that closes gaps in R6 and adds to the assembly in heterochromatin, most notably the Y chromosome. Even with long single-molecule reads, unequal read coverage across heterochromatic regions may cause assembly problems (Carvalho *et al.* 2016). Because assemblers typically use the top ∼30× longest reads for genome assembly, sex-linked regions may be undersampled. For example, some Y-linked regions are extremely underrepresented (*e.g.*, there are no reads from the third exon in *Ppr-Y* and only nine reads come from the second and third exons of *kl-3*). To reduce this potential bias, we assembled the heterochromatin and euchromatin separately and then combine these assemblies with each other and with published versions of the *D. melanogaster* genome (Figure 1). We first isolate a set of heterochromatin-enriched reads by mapping all Pacbio reads to the R6 reference and discarding reads mapping uniquely to the euchromatic genome (Figure 1A). Using this approach, we extracted ∼1.58 GB of sequence across 204,065 reads (12% of total reads) for assembly. With this small subset of reads, we are able to optimize parameters for repeat assembly, partially remedy assembly errors, and increase assembly contiguity. For Canu, we experimented with assembly conditions by varying bogart parameters (see Supplemental Methods). For Falcon, we experimented with the minimal overlap length in the string graph. For both methods, we identified parameter combinations that maximized assembly N50, total assembly length, and longest contig length; and without detectable misassemblies in Y-linked coding regions. We note that while assembly length and contiguity are often used to assess assembly quality, the most contiguous assemblies are not always correct (Khost *et al.* 2017). We therefore reconciled the assembled contigs from the two best versions of our heterochromatin-enriched and whole-genome assemblies sequentially, and finally, with the R6 assembly and another PacBio reference assembly (Figure 1, B and C, Figure S1, and Table S1; Chakraborty *et al.* 2016). Our final assembly contains major chromosome arms and mitochondrial sequences primarily from R6. The Y chromosome in our assembly, with the exception of three regions (*18HT*, and small parts of *Ppr-Y* and *kl-3*, totaling ∼164 kb) is *de novo* assembled (164 kb/14.5 Mb = 98.9%). We manually adjusted misassembled contigs and polished the final assembly for use in downstream analyses (Figure 1D, Figure S1, and Table S1). Our final reconciled genome has 200 contigs and is 155.6 Mb in total—a great improvement in assembly contiguity over R6 (143 Mb in 2,442 contigs; Table 1). The improvement is in both euchromatic and heterochromatic regions (Figures S2 and S3).

**Table 1:** Heterochromatin-enriched *D. melanogaster* assembly continuity statistics

Assembly		Summaries	
	No. of contigs	Total size	Contig N50
Whole genome			
GCF_000001215.4 (R6)	2442	143,726,002	21,485,538
Chakraborty *et al.**^a^*	767	149,071,519	21,492,213
GCA_002050065.1*^b^*	128	138,490,501	15,305,620
GCA_000778455.1*^b^*	789	164,080,454	13,636,574
This study	200	155,584,520	21,691,270
Y chromosome			
GCF_000001215.4 (R6)	261	3,977,036	81,922
This study	80	14,578,684	416,887

a Chakraborty *et al.* (2016).

b Berlin *et al.* (2015).

In addition to higher contiguity, our assembly also has a higher fraction of mapped reads than other assemblies (see Table 2). We quantified the number of putatively misassembled regions by aligning to the reference genome (R6; *e.g.*, Figures S2 and S3). Some of the differences between R6 and our assembly may correspond to misassemblies in R6. For example, *Mst77Y* and *Sdic* are misassembled in R6 (Krsticevic *et al.* 2015; Clifton *et al.* 2017). Our reconciliation process does not introduce a significant number of misassemblies (Table S4); however, we noticed that after polishing, the number of “local misassemblies” (85 bp–1 kb gaps) increases (from 1213 to 1346). Some of these “misassemblies” may represent polymorphisms within sequenced strain or misassemblies in R6 (Table S4). It is difficult to determine the correct assembly in repetitive regions; however, we do validate a subset of some genic regions on the Y chromosome where our assembly disagrees with R6 (see below and Table S3).

**Table 2:** *D. melanogaster* assembly assessment

Assemblies	Genome fraction (%)*^a^*	Duplication ratio*^a^*	Mapped (%)*^b^*	Properly paired (%)	Coverage > 10× (%)*^b^*	No. of misassemblies*^a^*	Mismatches per 100 kb*^a^*	Indel per 100 kb*^a^*
GCF_000001215.4 (R6)	NA	NA	97.89	94.18	98.82	NA	NA	NA
Chakraborty *et al.**^c^*	93.945	1.078	97.43	93.69	99.37	1048	61.61	36.93
GCA_002050065.1*^d^*	91.623	1.040	95.58	91.81	99.97	1382	78.08	23.99
GCA_000778455.1*^d^*	96.573	1.153	97.71	94.57	99.39	3508	171.30	38.52
This study	97.005	1.082	97.90	94.73	99.62	2408	110.61	17.54

a Relative to R6.

b Including reads from both Pacbio and Illumina.

c Chakraborty *et al.* (2016).

d Berlin *et al.* (2015).

Our new assembly fills all unassembled gaps in the euchromatic regions of the R6 major chromosome arms (one each on 2R, 3L, and 4; Figure S2 and Table S5), except for the histone cluster on chromosome 2L. Chromosome 4 had a predicted 17-kb gap in R6. In agreement with this predicted gap size, our new assembly inserts 17,996 bp in this gap with (AAATTAT)_n_ repeats and other AT-rich sequences. The gap on chromosome 2R was unsized; our assembly fills this gap with 4,664 bp consisting of 123-bp complex repeats. Interestingly, an annotated noncoding gene, CR44666, is located near the 2R gap in R6 and consists entirely of this 123-bp unit. In agreement with the predicted gap size of ∼7 kb on 3L, our new assembly inserts 6,157 bp containing one of four tandem copies of the 3S18/BEL transposons. Our assembly therefore places all euchromatic regions of the major chromosome arms on single contigs other than 2L.

We also made a marked improvement to heterochromatic regions [as defined by Hoskins *et al.* (2015)]. In total, we filled 25 out of 57 gaps in the R6 major chromosome scaffolds (Table S5). Of these gaps, 14 were located in transposon-dense regions; four were associated with complex repeats (two with *Responder*, one with *1.688* family repeats and one with a newly identified 123-bp unit), three were associated with 7-bp tandem repeats, and one was associated with ribosomal DNA (rDNA) repeats. One is a 17-kb deletion and the other two gaps involve complex rearrangements between R6 and our assembly that may represent scaffolding errors in R6. Our new assembly has ∼38.6 Mb of heterochromatin-enriched DNA across 193 contigs, whereas the R6 assembly has ∼26.7 Mb of heterochromatin-enriched DNA in 2432 contigs. Approximately 89% of the additional heterochromatic sequence in this assembly is from the Y chromosome (see below). We assigned some contigs based on their repeat content, *e.g.*, a 180-kb contig from chromosome *2* (contig 142). This contig terminates in (AATAACATAG)_n_ and (AAGAG)_n_ repeats mapping to cytological bands *h37* and *h38* (Garavís *et al.* 2015). Contig 142 extended an existing unmapped R6 scaffold (Unmapped_Scaffold_8_D1580_D1567), which contains a gene (*klhl10*) that maps to chromosome 2 (http://flybase.org/reports/FBgn0040038).

### Identifying Y-linked contigs

The estimated size of the Y chromosome is 40 Mb, however only ∼4 Mb is assembled and assigned to the Y chromosome in R6 (Hoskins *et al.* 2015). Our assembly pipeline based on PacBio reads circumvents the cloning steps associated with BAC-based sequencing, and results in a better representation of heterochromatin, including the Y chromosome. We developed an approach to identify and assign Y-linked contigs based on detecting male-specific sites using Illumina reads (Figure 1E). To validate our method to assign Y-linkage, we used contigs with a known location in R6 as benchmarks. Previous studies in mosquitos and *D. melanogaster* identified Y-linked contigs using the chromosome quotient (CQ): the female-to-male ratio of the number of alignments to a reference sequence (Hall *et al.* 2013). In *D. melanogaster*, this method has 76.3% sensitivity and 98.2% specificity (Hall *et al.* 2013). Our approach instead considers the number of male-specific regions (where the median per-site female-to-male ratio is 0) and is a better indicator of Y-linkage than CQ: among 14,116 10-kb regions in our assembly with known chromosomal location based on previous data (R6 assembly), we appropriately assigned 99.0% of Y-linked regions (714/721 regions; Figure S4). Only 1.5% of all regions that we assigned to the Y chromosome are not Y-linked in the R6 assembly (11/725 regions; Figure S4). Therefore, our method has both a higher sensitivity and specificity than previous methods. For the 11, 10-kb regions that may be false positives in our method, nine are from a centromeric scaffold (3Cen_31_D1643_D1653_D1791) and two are from the second chromosome telomeres. These regions may be misassigned in the R6 assembly because the centromeric scaffold has a Y-specific repeat, AAAT, (Wei *et al.* 2018) and telomeric transposons are found on all chromosomes and may vary within strains. The high sensitivity and specificity of this method also allows us to detect misassemblies. As we did not find inconsistencies in this ratio across contigs, we are unlikely to have many misjoins between Y-linked sequences and other chromosomes. We used our method to assign 14.6 Mb to the Y chromosome across 106 contigs (N50 = 415 kb; Table 1). The distribution of Pacbio read depth across Y-linked regions in our assembly is more normally distributed than Y-linked regions in the R6 assembly (Figure S5). Because ∼80% of the 40-Mb Y chromosome consists of tandem repeats (Lohe *et al.* 1993), this is likely near the maximum amount of Y-linked sequence we can expect to identify with current sequencing technology.

### Improving known Y-linked gene annotations

**Figure 2:**
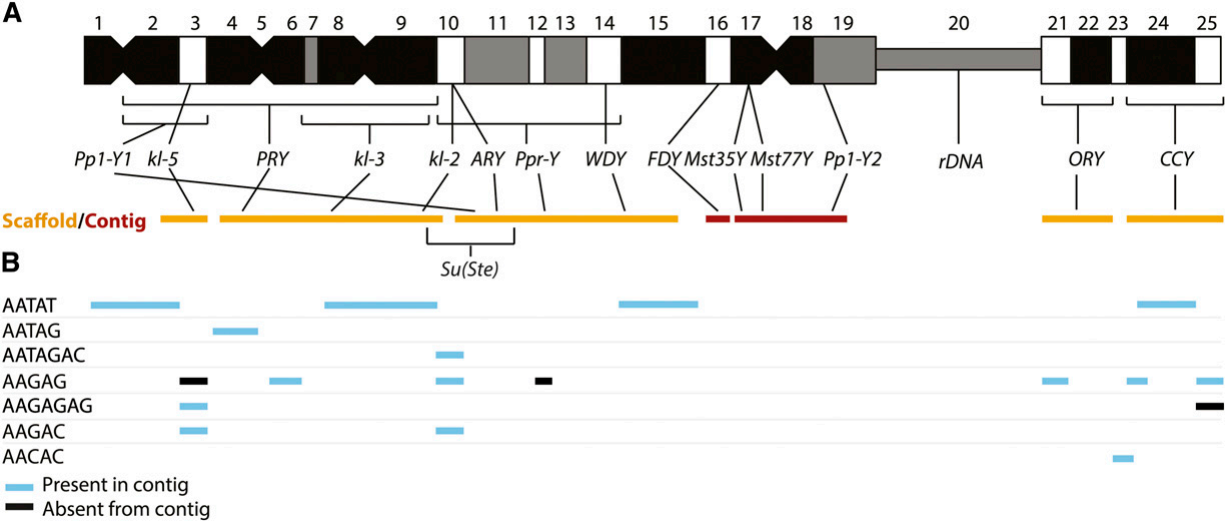
Schematic of Y chromosome organization. (A) The Y chromosome is organized into 25 cytological bands. The position of the Y-linked genes is shown based on deletion mapping (Carvalho *et al.* 2000, 2001; Vibranovski *et al.* 2008). The major scaffolds (orange bars) and contigs (dark red bars) that span each Y-linked gene, from left to right, are as follows: Y_scaffold6, Y_scaffold7, Y_scaffold4, Y_Contig10, Y_Contig2, Y_scaffold5, and Y_scaffold3. Note that scaffolds contain gaps. (B) The approximate cytological location of large blocks of simple tandem repeats (Bonaccorsi and Lohe 1991) agrees with the organization of our scaffolds and contigs: blue bars indicate that a block of satellite appears in that contig/scaffold, and black bars indicate that a block of repeats is missing from that contig/scaffold. Note that missing repeats may fall entirely in the gaps in our scaffolds, and potential cross-hybridization between AAGAG and AAGAGAG might explain the three discrepancies between our assembly and the cytological map.

The gene order and orientation of Y-linked genes in our assembly is consistent with previous mapping data (Figure 2; Carvalho *et al.* 2000; Carvalho *et al.* 2001; Vibranovski *et al.* 2008) using Y chromosome deletions, except for *Pp1-Y1*. We found high-quality mapped reads supporting the bridge between *Pp1-Y1* and the *Su(Ste)-PCKR* family at h14-16 (see Figure S6). Unfortunately, we cannot distinguish whether this difference is due to a misassembly or strain variation. We found splice site errors in three previous Y-linked gene models: the intron between sixth and seventh exons of *kl-2* is missing, *kl-5* has four additional introns (one in the first, two in the fifth, and one in the 17th exons of the R6 annotation; Table S6), and *CCY* has one additional intron (in the sixth exon of the R6 annotation; Table S6). We also found partial duplications of exons in *kl-3*, *ORY*, *Ppr-Y*, and *WDY* (Table S7). Each of these duplications, except *ORY*, exists on unannotated regions of the R6 assembly. In the R6 assembly, *CCY* and *kl-3* contain misassembled sequences in sixth and third exon coding regions, respectively. We therefore corrected the misassemblies in the R6 Y-linked coding regions based on our assembly and PCR validation (Table S3).

### Y-linked gene duplications

We identified 13 independent duplications to the Y chromosome from other chromosomes, seven of which we identify as Y-linked for the first time. Eleven of these duplications exist in multiple copies on the Y chromosome (Table 3). We also identified a new Y-linked gene, *CG41561*, located on an unmapped contig (211000022280328) in the R6 assembly (Mahajan and Bachtrog 2017). Among the 13 duplications, we found that the Y-linked copies of *Hsp83*, *Mst77F* (*Mst77Y*), and *vig2* (*FDY*) are still expressed in testes (Fragments Per Kilobase Million >5 in at least one data set; Table S8); however, the expressed Y-linked *Hsp83* contains a premature stop codon and a TE insertion. Therefore, outside of *Mst77Y* and *FDY*, we do not have evidence for their function (Krsticevic *et al.* 2010, 2015). Interestingly, these duplications seem to be clustered on the Y chromosome: six of duplications are on Y_scaffold4 and five of the duplications are on Y_Contig2 (Table 3). Y_scaffold4 and Y_Contig2 are from the cytological divisions *h10-15* and *h17-18*, respectively (Figure 2). Additionally, *FDY* (Y_Contig10) maps to *h15-h20* (Krsticevic *et al.* 2015). Therefore, 12 out of 13 duplications are located between *h10-h20* (11 out of 25 Y-linked cytological bands), suggesting that the pericentromere of the Y chromosome (defined here as *h10-h20*) is enriched for duplicated genes in *D. melanogaster* (Fisher’s exact test, *P* = 0.005).

**Table 3:** Translocations to the Y chromosome from the autosomes and X chromosome

Parent genes	Parent	Y copy no.	Location of duplication on Y	Source	Name	Reference
*Gs1l*	2L	2	Y_scaffold4	DNA	NA	Tobler *et al.* (2017)
*smt3*	2L	5	Y_scaffold4, Y_Contig140, Y_Contig23	RNA	NA	NA
*ProtA*	2L	9	Y_Contig2, Y_Contig6, Y_Contig104	DNA	*Mst35Y*	Mendez-Lago *et al.* (2011)
*Hsp83*	3L	6	Y_scaffold4	RNA	NA	NA
*velo*	3L	70	Y_Contig2, Y_Contig6, Y_Contig104	unknown	NA	NA
*Pka-R1*, *CG3618*, *Mst77F*	3L	15,17,18	Y_Contig2	DNA	*Mst77Y*	Krsticevic *et al.* (2010)
*Dbp80*	3L	1	Y_scaffold6	DNA	NA	NA
*fru*	3R	6	Y_scaffold4	unknown	NA	NA
*CG5886*	3R	2	Y_scaffold4	unknown	NA	NA
*vig2,Mocs2,Clbn,Bili*	3R	1,1,7,1	Y_Contig10	DNA	*FDY*	Carvalho *et al.* (2015)
*Tctp*	3R	1	Y_scaffold4	unknown	NA	NA
*CR43975*	3R	78	Y_Contig2, Y_Contig4, Y_Contig6, Y_Contig104, Y_Contig22	DNA	NA	Tobler *et al.* (2017)
*CG12717*, *ade5*	X	214,33	Y_Contig2, Y_Contig6, Y_Contig104	DNA	NA	Mendez-Lago *et al.* (2011)
*Unknown*	*^a^*	1	Y_Contig74	NA	*CG41561*	Mahajan and Bachtrog (2017)

a CG41561 has no known homolog and is located on Unmapped contig 211000022280328 in R6.

### Repeat content in Y-linked contigs

**Figure 3:**
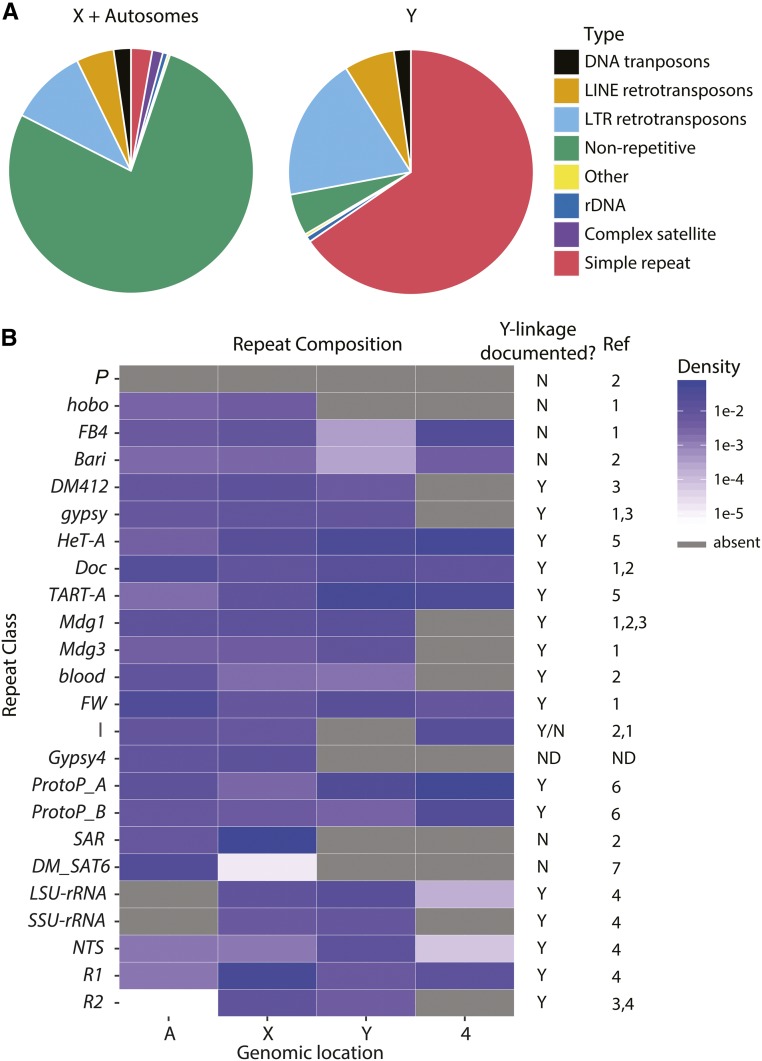
Repeat composition on the Y chromosome compared to the rest of the genome. (A) The major repeat class composition on Y-linked contigs and all other contigs in our assembly (from the X and autosomes). (B) A comparison of complex repeats and transposable elements between autosomes, X, Y, and fourth chromosomes. We indicate the presence/absence (Y/N, respectively) of repeat classes for which cytological and/or Southern hybridization data exists in the literature. *I* elements have conflicting reports of Y-linkage in the literature. References: (1) Carmena and Gonzalez (1995); (2) Pimpinelli *et al.* (1995); (3) Junakovic *et al.* (1998); (4) Ritossa and Spiegelman (1965); (5) Agudo *et al.* (1999); (6) Balakireva *et al.* (1992); (7) Abad *et al.* (1992).

Cytological observations indicate that the Y chromosome is highly enriched for repetitive sequences (Lohe *et al.* 1993; Carmena and Gonzalez 1995; Pimpinelli *et al.* 1995); however, there have not been attempts to document this at the sequence level. We used our assembly to identify repetitive elements across the Y chromosome. Consistent with previous studies, we find that the Y chromosome is enriched for rDNA and their intergenic repeats (IGS) (Ritossa and Spiegelman 1965; Figure 3A and Table S9). The rDNA are located across 54 scaffolds/contigs, including 1 Y-linked scaffold, 12 Y-linked contigs, 2 X-linked contigs, and 39 unknown contigs (Table S9). We identified 56 copies of 18S rDNA, 238 copies of 28S rDNA, and 721 copies of IGS repeats on the Y chromosome. Long terminal repeat (LTR) transposons and long interspersed nuclear elements (LINEs) contribute 53 and 19% of the total sequence, respectively, in our Y-linked contigs (Figure 3A). We assume that most of the unassembled parts of the Y chromosome are simple tandem repeats (Lohe *et al.* 1993). Based on this assumption, we estimate that 65% of the 40-Mb Y chromosome is simple tandem repeats, and LTR and LINE elements comprise 18 and 7% of the total 40-Mb Y chromosome, respectively. Compared to the rest of the genome, the Y chromosome has a 1.4- to 1.8-fold enrichment of retrotransposons (10.2% of LTR and 5.0% of LINE for the rest of the genome), while DNA transposon content is similar among chromosomes (2.3% on Y and 2.2% for the rest of the genome; Figure 3A). The Y chromosome is enriched for retrotransposons over DNA transposons even when compared to other heterochromatic genomic regions (Figure S7).

Previous studies predicted the repeat composition of the Y chromosome based on the presence/absence of *in situ* hybridization (ISH) signals on mitotic chromosomes (Carmena and Gonzalez 1995; Pimpinelli *et al.* 1995). Our assemblies recapitulate these ISH results. For example: *P*, *hobo*, *FB4*, and *Bari-1* are nearly absent from the Y chromosome (<3.5 kb of total sequence), while *Dm412*, *Gypsy*, *HetA*, *Doc*, *TART*, *Mdg1*, *Mdg3*, *blood*, and *FW* have at least 14 kb of sequence on the Y chromosome (Figure 3B and Table S9; Carmena and Gonzalez 1995; Pimpinelli *et al.* 1995; Junakovic *et al.* 1998; Agudo *et al.* 1999). There are conflicting reports on the presence/absence of Y-linked *I* elements in the literature (Carmena and Gonzalez 1995; Pimpinelli *et al.* 1995). We do not see evidence of Y-linked *I* elements in our assembly. Other transposons also appear to be absent from the Y chromosome, *e.g.*, *gypsy4* (Table S9; Figure 3B). Since *I*-element–mediated dysgenesis only occurs in females (Bucheton *et al.* 1976), it is possible that this element is inactive in the male germline and therefore rarely has the opportunity to invade Y chromosomes. We suggest that the sex-specific activity of TEs may contribute to their genomic distribution.

Tandem repeats are also enriched on Y chromosomes (∼65% on the Y chromosome compared to 2.8% on the other chromosomes; Lohe *et al.* 1993). Approximately 5% (742,964 bp) of our Y-linked sequences correspond to tandem repeats. We assume that this is a gross underestimate of tandem repeat abundance, but nevertheless helps lend insight into the repeat content and organization of the Y chromosome. Our assembly agrees with most previous cytological and molecular evidence of Y chromosome simple tandem repeat content (Figure 2; Bonaccorsi and Lohe 1991). Among 32 known Y-linked simple repeats, 20 appear in our Y-linked contigs (Table S10; Bonaccorsi and Lohe 1991; Jagannathan *et al.* 2017; Wei *et al.* 2018). The repeats that we do not find may be sequence variants of abundant repeats (*e.g.*, we detect AAAAC and AAAGAC but not AAAAAC or AAAAGAC), not perfectly in tandem, or part of a more complex repeat (*e.g.*, AAGACAAGGAC is part of AAGACAAGGAAGACAAGGACAAGACAAGGAC; Table S10). Although we recover only ∼60% of known Y-linked repeats (based on Illumina data, Wei *et al.* 2018; or ISH, Bonaccorsi and Lohe 1991; Jagannathan *et al.* 2017), our new assembly including genes and transposable elements provides the most detailed view of Y chromosome organization.

### Evolution of the *crystal-Stellate* gene family

**Figure 4:**
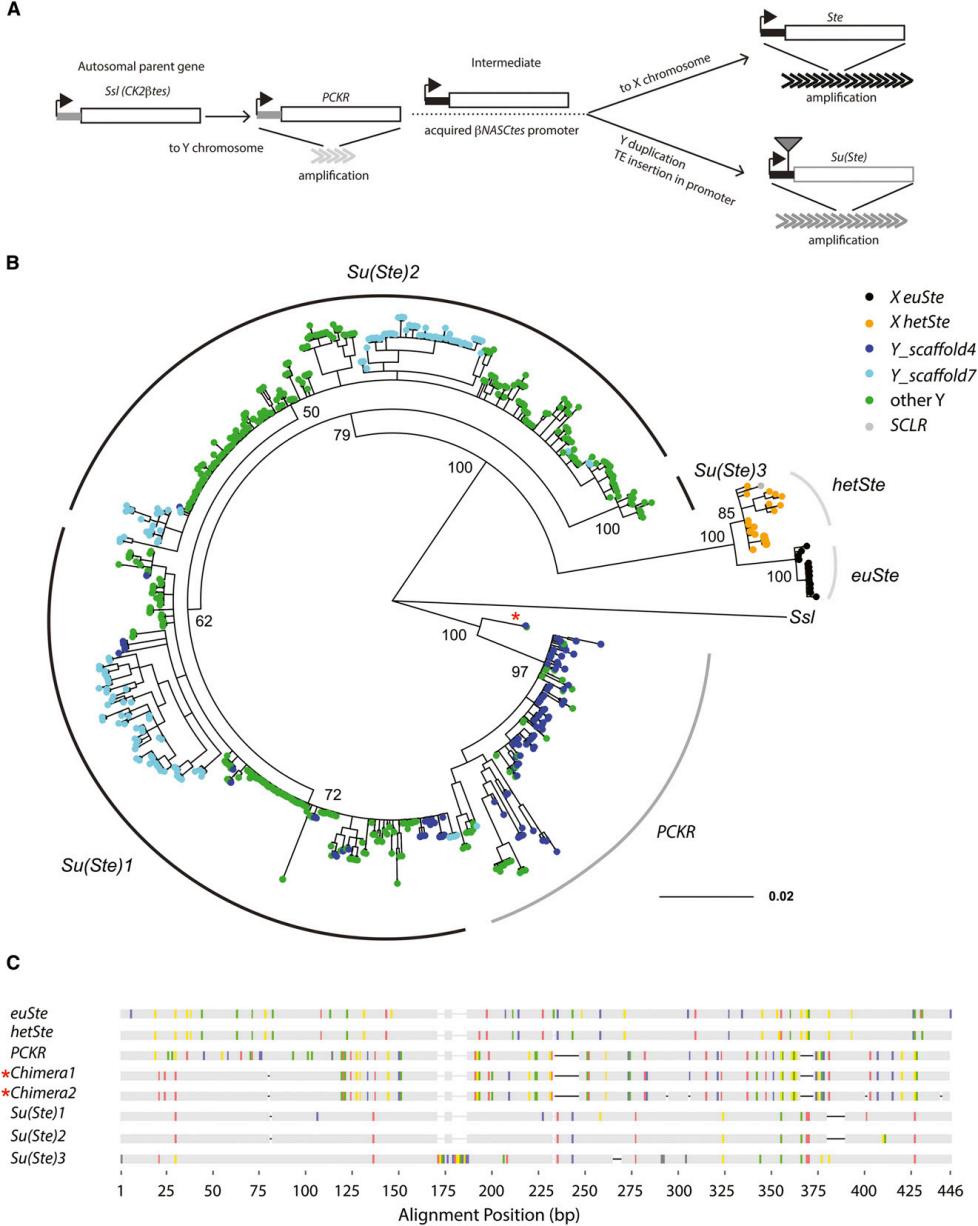
Evolution of the *Cry-Ste* family. (A) The evolutionary history of *Cry-Ste* family in *D. melanogaster* [modified from Usakin *et al.* (2005)]. (B) A Bayesian phylogenetic tree constructed with 606 full-length copies of genes in the *Cry-Ste* family including *Ssl* (parent gene) as the outgroup. Tip colors represent the location of genes in our assembly. Posterior node confidence is shown for a subset of the primary nodes separating repeat types. SCLR is a nonfunctional variant of *Ste*. (C) The alignment of representative repeats for heterochromatic Ste (*hetSte*), euchromatic Ste (*euSte*), *PCKR*, three main variants of *Su*(*Ste*), and two chimeric genes are shown (also indicated with red * in tree). Vertical colored lines indicate where base changes (red = A; yellow = G; green = T; blue = C; gray = missing) occur and dashes indicate indels.

The multicopy *crystal-Stellate* (*cry-Ste*) gene family is thought of as a relic of intragenomic conflict between X and Y chromosomes [reviewed in Bozzetti *et al.* (1995), Hurst (1996), Malone *et al.* (2015)]. *Stellate* (*Ste*) is an X-linked multicopy gene family whose expression is controlled by the Y-linked *Suppressor of Stellate* [*Su*(*Ste*)] locus via an RNA interference mechanism (Nishida *et al.* 2007). If left unsuppressed, *Ste* expression leads to the accumulation of crystals in primary spermatocytes of the testes and male sterility (Bozzetti *et al.* 1995). This multicopy gene family has a complicated evolutionary history (Kogan *et al.* 2000). *Ste* and *Su(Ste)* are recent duplications of the autosomal gene *Su(Ste)-like* (*Ssl* or *CK2*β) with a testis-specific promoter from casein kinase subunit 2 (Kogan *et al.* 2000). Following the initial duplication of *Ssl* to the Y chromosome, members of this gene family expanded and duplicated to the X chromosome (Figure 4A). All sex-linked members of this gene family exist in multiple copies. The X-linked copies and Y-linked copies amplified independently, perhaps driven by sex chromosome conflict (Kogan *et al.* 2012). We used our assembly to study the evolution of this interesting gene family and patterns of gene conversion on the Y chromosome. We found 666 copies of genes in the *cry-Ste* family: 37 on the X chromosome, 627 on the Y chromosome, and two from an unknown region. We detect more Y-linked copies than were previously estimated (200–250 complete copies) using Southern blotting (McKee and Satter 1996). We found a clade of 122 Y-linked genes that are from an ancestral duplication of *Ssl* and fall as an outgroup to *Ste* and *Su(Ste)* (Figure 4B). These copies, originally identified in a Y-derived BAC, are designated as pseudo-*CK2*β repeats on the Y chromosome (*PCKR*s) and have the ancestral promoters (Danilevskaya *et al.* 1991; Usakin *et al.* 2005). However, there is very little expression among the 107 copies of *PCKR* (total Fragments Per Kilobase Million <3 from *CR40947* and MSTRG.17120.1; Table S8). *Ste* copies appear in both the X heterochromatin and euchromatin (hereafter referred to as *hetSte* and *euSte*, Livak 1984; Shevelyov 1992). In addition to the 13 previously assembled copies of *euSte* (cytological divisions 12E1 to 12E2), we found an additional 20 copies of *Ste* located on two X-linked contigs (17 on Contig5 and 3 on X_9), corresponding to functional *hetSte* copies and a region with *Stellate* genes, *Copia*-like retrotransposons; LINE elements, and rDNA fragments (SCLR; Nurminsky *et al*. 1994; Tulin *et al*. 1997). The three Stellate repeats in the SCLR on the contig X_9 were present but not annotated in the R6 assembly and were located proximal to *hetSte*. We assembled 17 *hetSte* in a single 500-kb contig, where two *hetSte* loci (5 and 12) are separated by *BATUMI* and rDNA sequences. However, previously published data using restriction maps and Southern blotting suggests that *hetSte* are organized into three loci (with ∼14, 3, and 4 copies) separated by *BATUMI* and rDNA (Tulin *et al.* 1997). Our phylogenetic analysis reveals that *Ste* in SCLR and *hetSte* are clustered, suggesting that *hetSte* and *euSte* amplified independently or experience concerted evolution (Figure 4B).

The 627 *Su*(*Ste*) and *PCKR* copies are spread across 10 and 3 Y-linked contigs, respectively. These repeats primarily occur in tandem and are flanked by different transposon sequences, including *1360*, *Gypsy12*, and the telomere-associated transposons, *HeT-A*, *TART*, and *TAHRE*. Previous studies suggested that the acquisition of *1360* in *Su*(*Ste*) may have been an important step in *Su*(*Ste*) evolving a Piwi-interacting RNA (piRNA) function to suppress *Ste* (Usakin *et al.* 2005). *HeT-A* colocalizes with *Ste*-like sequences in the BAC Dm665 (Danilevskaya *et al.* 1991). We found that the S*te*-like sequences in Dm665 are PCKRs and are located proximal to *Su*(*Ste*), between *WDY* and *Pp1-Y1*. Consistent with BAC data and our assembly, this region is also enriched for telomeric sequences (based on ISH, Figure S6; Traverse and Pardue 1989; Abad *et al.* 2004). Interestingly, we found two chimeric copies of *PCKR* and *Su*(*Ste*) (Figure 4C), suggesting intergenic gene conversion occurred between these genes. Previous studies hypothesized that gene conversion homogenizes *Su*(*Ste*) clusters, but these studies were only based on restriction maps or a few variants (Balakireva *et al.* 1992; McKee and Satter 1996). We investigated the rate of gene conversion on the Y chromosome using 107 copies of *PCKR* and 406 copies of *Su*(*Ste*) after removing fragments smaller than 280 bp. We detected evidence of recombination at both *PCKR* (per 857-bp locus: Rmin = 2 and ρ = 2.67; *c_g_* = 2.9 × 10^−5^ events per site, per generation) and *Su*(*Ste*) (per 1203-bp locus: Rmin = 1 and ρ = 4.04; *c_g_* = 8.3 × 10^−6^ events per site, per generation). Since there is no recombination via crossing over, we estimate the Y-linked gene conversion rate to be 0.8–5 × 10^−5^ events per site, per generation. We also used estimates of similarity among repeats within each gene family to estimate gene conversion rates (Supplemental Methods; *c_g_*). Assuming a mutation rate of 2.8 × 10^−9^ per site per generation (Keightley *et al.* 2014), we estimate the rate of gene conversions per site per generation to be 2.1 × 10^−5^ and 1.5 × 10^−4^ for *PCKR* and *Ste*, respectively. These rates are ∼10^3^–10^4^ times higher than gene conversion rates on the autosomes and X chromosome (Comeron *et al.* 2012; Miller *et al.* 2012, 2016), and surprisingly similar to the rate observed in mammalian Y and bird W chromosomes (Repping *et al.* 2003; Backström *et al.* 2005; both based on *c_g_*). Rmin and linkage disequilibrium (LD)-based estimators may underestimate the true gene conversion rate because both recent amplification and selection could decrease variation among copies and cause us to miss recombination events. On the other hand, we likely overestimate the gene conversion rate based on similarity among copies for the same reasons. With both approaches, our data suggest high rates of intrachromosomal gene conversion on Y chromosomes. Recombination may also occur between the X and Y chromosomes: of the 116 variant sites in *Ste*, 62 of the same variants are found at the homologous positions in *PCKR* and/or *Su*(*Ste*). It will be important to further explore rates of Y-linked gene conversion using multiple strains of *D. melanogaster*. Higher gene conversion rates in Y-linked multicopy gene families may be important for the evolution of Y-linked genes.

## Discussion

Heterochromatic sequences can contain important genetic elements (*e.g.*, Gatti and Pimpinelli 1992) but tend to be underrepresented in genome assemblies. Single-molecule real-time sequencing is making strides toward achieving complete assemblies of complex genomes (Huddleston *et al.* 2014; Chaisson *et al.* 2015); however, densely repetitive regions still present a significant assembly challenge that often requires manual curation (Krsticevic *et al.* 2015; Clifton *et al.* 2017; Khost *et al.* 2017). Uneven read coverage across the genome and lower read coverage in heterochromatic regions likely cause problems with genome assembly (Krsticevic *et al.* 2015; Chang and Larracuente 2017; Khost *et al.* 2017). Our assembly approach is based on the *in silico* enrichment of heterochromatic reads, followed by the targeted reassembly of heterochromatic regions, and finally, a reconciliation between whole-genome and heterochromatin-enriched assemblies. This approach helped fill gaps, fix errors, and expand the *D. melanogaster* reference assembly by 11.9 Mb (8% more sequence than the latest release, R6). Approximately 89% of the additional sequence in our assembly is from the Y chromosome, allowing us to get a detailed view of Y chromosome organization. Despite these improvements, we are still missing some Y-linked regions and some required manual correction. Assemblers filter reads when they appear chimeric or where pairs of reads disagree about overlaps. Canu and Falcon tend to disagree about the organization of some highly repetitive sequences (*e.g.*, *Rsp*, Khost *et al.* 2017; *Sdic*, Clifton *et al.* 2017; and *Mst77Y*, Krsticevic *et al.* 2015). Our approach does not completely remedy this problem, as we also identified errors in our preliminary assemblies that required manual correction. For these misassembled regions, Falcon and Canu arrive at different sequence configurations (*e.g.*, we found 20 copies of *Mst77Y* in the Canu assembly and 14 copies in the Falcon assembly). To resolve these differences, we leveraged evidence from ISH studies and known gene structures to identify and reconcile differences between the assemblies. Our results suggest that merging multiple assemblies and examining discordant regions using independent evidence is instrumental in assembling complex genomes.

Our biggest improvement to the assembly was on the Y chromosome, which has an unusual composition: its ∼20 genes are interspersed among ∼40 Mb of repetitive elements (Ritossa and Spiegelman 1965; Lohe *et al.* 1993; Carmena and Gonzalez 1995; Pimpinelli *et al.* 1995; Abad *et al.* 2004). Natural variation among *D. melanogaster* Y chromosomes can have wide effects on genome function and organismal fitness (*e.g.*, Carvalho *et al.* 2000; Vibranovski *et al.* 2008; Paredes *et al.* 2011; Francisco and Lemos 2014; Kutch and Fedorka 2017; Wang *et al.* 2017). The extremely low nucleotide diversity of Y-linked genes (*e.g.*, Zurovcova and Eanes 1999; Larracuente and Clark 2013; Morgan and Pardo-Manuel de Villena 2017) suggests that the Y-linked functional variation likely maps to the non-genic regions. The Y chromosome is a strong modifier of position effect variegation, a phenomenon that results in the stochastic silencing of euchromatic reporters caused by the spreading of heterochromatin (Karpen 1994; Elgin 1996; Wakimoto 1998). Y chromosomes may act as heterochromatin sinks, where extra Y-linked heterochromatin can titrate available heterochromatin-binding proteins away from other genomic locations. This may explain how genetic variation in Y-linked heterochromatin affects global gene expression (Henikoff 1996; Francisco and Lemos 2014; Brown and Bachtrog 2014 and unpublished data). Alternatively, variation in Y-linked loci that generate small RNAs may have widescale effects on chromatin organization (Zhou *et al.* 2012). These effects are difficult to tease apart without having a detailed view of Y chromosome sequence and organization. Our study discovered features of the Y chromosome that may relate to its interesting biology. Variation in Y-linked heterochromatin may affect the amount of silent chromatin marks in transposons (Brown and Bachtrog 2014 and unpublished data), perhaps contributing to the higher rate of TE activity in males. We show that RNA transposons are generally overrepresented on the Y chromosome. It is possible that the overrepresentation of Y-linked retrotransposons is due to their increased activity in males: the Y chromosome heterochromatin sink effect may lead to reduced transcriptional silencing of TEs. In contrast to DNA transposons, the movement of retrotransposons is transcription dependent and therefore may result in differences in activity between the sexes. If the Y chromosome behaves as a sink for heterochromatin proteins, then we may expect the overrepresentation of RNA transposons to be a universal feature of Y chromosomes. Alternatively, differences in DNA repair or nonhomologous recombination might lead to the differential accumulation of DNA and retrotransposons on the Y chromosome compared to the rest of the genome.

Y-linked structural variations can affect genome-wide gene regulatory variation in flies [*e.g.*, *Su*(*Ste*) and *rDNA*; Lyckegaard and Clark 1989; Zhou *et al.* 2012] and male fertility in mammals (Reijo *et al.* 1995; Vogt *et al.* 1996; Sun *et al.* 2000; Repping *et al.* 2003; Morgan and Pardo-Manuel de Villena 2017). We find a large amount of gene traffic to the *D. melanogaster* Y chromosome from elsewhere in the genome. While estimates of interchromosomal duplications between the X and major autosomal arms range from ∼3 (Bhutkar *et al.* 2007) to 7 (Han and Hahn 2012) on the *D. melanogaster* branch, we find at least 10 interchromosomal duplications to the Y chromosome. This observation is similar to other studies across taxa (Koerich *et al.* 2008; Hall *et al.* 2013; Hughes and Page 2015; Mahajan and Bachtrog 2017; Tobler *et al.* 2017). Our Y chromosome assembly provides new insights into the organization and mechanisms behind these duplications. For example, we found that most new translocations are DNA based and clustered in the Y pericentromic heterochromatin. The Y chromosome heterochromatin appears to be distinct from other heterochromatic regions of the genome, with properties that vary along the length of the chromosome (Wang *et al.* 2014). We hypothesize that the Y chromosome pericentromeric heterochromatin may be more accessible than other regions of the chromosome. If so, the increased accessibility may affect transcriptional activity and make these regions more prone to double-strand breaks (DSBs) that would facilitate structural rearrangements. Therefore, Y-linked pericentromeric chromatin may be more permissive to transcription compared to the rest of the chromosome allowing for natural selection to retain insertions that result in functional products. This may provide insights into how new Y-linked genes gain testis-specific functions. Notably, most Y-linked translocations are DNA-based and therefore involve DSB repair. Without a homolog to provide a template for DSB repair, microhomology-mediated end-joining of nonhomologous sequences may lead to insertions in the Y chromosome. DSB repair may also result in tandem duplications that contribute to the observed copy number variation in Y-linked genes. We discovered that most of the recent translocations to the Y chromosome exist in multiple copies (Table 2), suggesting that the tandem duplication rate may also be higher in the pericentric regions. However, most of these newly acquired genes are pseudogenized and are likely not constrained by natural selection. Many functional Y-linked genes are at least partially duplicated. Most essential Y-linked genes (*kl-2*, *kl-3*, *kl-5*, and *ORY*) have larger introns (>100 Kb), with some introns reaching megabases in size (Kurek *et al.* 2000; Reugels *et al.* 2000). For genes with large overall sizes, complete gene duplications are less likely. In contrast, some functional genes [*e.g.*, *rDNA*, *Mst77-Y*, and *Su*(*Ste*)] exist in multiple copies and are sensitive to gene dosage (Lyckegaard and Clark 1989; Zhou *et al.* 2012; Kost *et al.* 2015). A high duplication rate on the Y chromosome may therefore facilitate the evolution of Y-linked gene expression.

In mammals, some Y-linked genes have amplified into tandem arrays and exist in large palindromes (*e.g.*, Rozen *et al.* 2003; Hughes *et al.* 2012; Soh *et al.* 2014). Gene conversion within these palindromes may be important for increasing the efficacy of selection on an otherwise nonrecombining chromosome (Charlesworth 2003; Rozen *et al.* 2003; Connallon and Clark 2010). Interestingly, the largest gene families in the *D. melanogaster* genome, outside of the rDNA and histone clusters, are the Y-linked genes *Su*(*Ste*) and *PCKR*. We inferred a higher rate of gene conversion in both *PCKR* and *Su*(*Ste*) than the rest of the genome, and similar to the rate observed in mammalian Y chromosome (Rozen *et al.* 2003). However, our estimates do not consider recent selection or amplification of *PCKR* and *Su*(*Ste*). The elevated Y-linked gene conversion rates may be a consequence of having more highly amplified gene families than other genomic locations. Alternatively, the Y chromosome may have evolved distinct patterns of mutation because it lacks a homolog: low copy number Y-linked genes also have relatively high rates of gene conversion in *Drosophila* (Kopp *et al.* 2006) and humans (Rozen *et al.* 2003). Gene conversion between members of Y-linked multicopy gene families may counteract the accumulation of deleterious mutations through evolutionary processes such as Muller’s ratchet [reviewed in Charlesworth and Charlesworth (2000), Charlesworth (2003), Rozen *et al.* (2003), Connallon and Clark (2010)]. If so, then we might expect high gene conversion rates to be a common feature among Y chromosomes.
